# Surgical Excision of Benign Papillomas Diagnosed with Core Biopsy: A Community Hospital Approach

**DOI:** 10.1155/2011/679864

**Published:** 2011-11-30

**Authors:** Eka Rozentsvayg, Kristen Carver, Sunita Borkar, Melvy Mathew, Sean Enis, Paul Friedman

**Affiliations:** Carol W. and Julius A. Rippel Breast Center, Morristown Memorial Hospital, Morristown, NJ 07962, USA

## Abstract

Our goal was to assess the value of surgical excision of benign papillomas of the breast diagnosed on percutaneous core biopsy by determining the frequency of upgrade to malignancies and high risk lesions on a final surgical pathology. We reviewed 67 patients who had biopsies yielding benign papilloma and underwent subsequent surgical excision. Surgical pathology of the excised lesions was compared with initial core biopsy pathology results. 54 patients had concordant benign core and excisional pathology. Cancer (ductal carcinoma in situ and invasive ductal carcinoma) was diagnosed in five (7%) patients. Surgery revealed high-risk lesions in 8 (12%) patients, including atypical ductal hyperplasia, atypical lobular hyperplasia, and lobular carcinoma in situ. Cancer and high risk lesions accounted for 13 (19%) upstaging events from benign papilloma diagnosis. Our data suggests that surgical excision is warranted with core pathology of benign papilloma.

## 1. Introduction

While considered a benign diagnosis, intraductal papillomas of the breast diagnosed with percutaneous core biopsy have traditionally been managed by complete surgical excision to carefully examine the entire papilloma and adjacent breast. Reasons to excise percutaneously diagnosed papillomas include difficulties in pathologic interpretation (particularly in the distinction of benign papillomas from papillary carcinomas), association with adjacent high risk lesions or cancers, and the premalignant potential of these lesions [1]. 

Over the past ten years though, some studies have suggested that benign papillomas diagnosed with core biopsy may be clinically followed rather than surgically excised. The management of intraductal papillomas has thus become controversial with some advocating surgical excision of all lesions despite benign pathologic features, whereas others excise only those specimens with atypical papillary features [2].

We sought to examine the degree of pathologic concordance between percutaneous core biopsy yielding benign papilloma and final excisional histology, thus contributing to guidelines regarding management of benign papillary lesions diagnosed on core biopsy. Because our study was conducted at a community hospital, we seek to translate our results, conclusions, and their implications to the general population.

## 2. Materials and Methods

The study was approved by the institutional review board of Atlantic Health at Morristown Medical Center, Morristown, NJ. In our retrospective observational study we evaluated 81 consecutive patients that were diagnosed with benign papilloma by core biopsy at Carol W. and Julius A. Rippel Breast Center from January 2006 to January 2010. 

The pathologies of 81 patients were determined to meet the inclusion criteria of a benign, concordant papilloma by percutaneous core biopsy. Any papillary lesion with atypical features diagnosed on core biopsy was excluded from the study. Our investigation was to assess upgrade rate of purely benign papillary lesions diagnosed on core biopsy. All lesions were discovered by screening mammogram and subsequent diagnostic workup with mammography and ultrasound. The 67 lesions were identified in 67 individual women. The biopsy methods included stereotactic core biopsy, ultrasound-guided core biopsy, and MRI-guided core biopsy. The stereotactic core biopsies were performed on a LoRad table with a SenoRx 11 Gauge vacuum-assisted core biopsy needle. The ultrasound-guided biopsies were performed utilizing a GE ultrasound for visualization of the lesion and a BARD monopty 14G needle for core biopsy. MRI-guided biopsies were performed using a 1.5 T GE Signa Excite system with a 7CH Invivo biopsy breast array coil for visualization and a 10 Gauge SenoRx EnCore vacuum-assisted core needle for biopsy.

All core biopsy results were compared with the final pathology upon surgical excision. Of the 81 patients, 67 women underwent subsequent surgical excision of papilloma, while 7 patients elected not to undergo the excision and seven were lost to follow up. Histopathologic examination was performed on the 67 lesions from the patients who underwent percutaneous core needle biopsy and subsequent excision of the benign papillomas.

## 3. Results and Discussion

Fifty-four of the sixty-seven lesions that underwent surgical excision were benign, accounting for 81% of the final pathology. Cancer was found on excision in five of the sixty-seven lesions which underwent surgical excision, accounting for 7% of the pathology. This resulted in 7% upstaging rate from benign papilloma to carcinoma upon final surgical excision. Of the diagnosed cancer, 2 out of 5 (40%) were ductal carcinoma in situ (DCIS) and 3 out of 5 (60%) were invasive ductal carcinoma. In addition, high-risk lesions including atypical ductal hyperplasia (ADH), atypical lobular hyperplasia (ALH), and lobular carcinoma in situ (LCIS) were identified in 8 out of 67 lesions upon subsequent surgical excision, accounting for 12% of the final pathology. Of the eight high-risk lesions, 75% were ADH, 12.5% were ALH, and 12.5% were LCIS. Overall, there was discordance of 19% between percutaneous core biopsy and surgical excisional pathology (Table 1).

There is a general consensus that papillomas with atypical features diagnosed on percutaneous core needle biopsy warrant surgical excision [3–6]. However, controversy persists regarding management of papillomas without atypical features. Across multiple series in the literature, a significant number of core biopsies that have yielded benign or bland papillomas resulted in a considerable upgrade rate to atypia and/or cancer upon surgical excisional pathology [6–11]. Many investigators have suggested that all papillary lesions diagnosed with percutaneous core biopsy, regardless of the presence or degree of atypia, should be surgically excised [6–12]. Alternatively, others have argued [2–5, 13, 14] that, while atypical papillary lesions found at core biopsy should be surgically excised, those lesions which have no atypical features at core biopsy may be managed conservatively with clinical and mammographic or sonographic followup. Therefore, there is uncertainty in management guidelines for the patients diagnosed with benign papillary lesion on percutaneous core biopsy.

Review of the literature reveals that many of the published studies in the past decade which investigated the upgrade rate of benign papillomas found on percutaneous core biopsies were limited by small sample size and at least fourteen studies have included less than 50 patients (Table 2). Four out of these studies, those of Ahmadiyeh, Sydnor, Renshaw and Agoff, each independently concluded that benign papillomas at core biopsy are infrequently associated with malignancy at excision (0–3%) and may be followed clinically and mammographically. Three out of these four studies [2, 3, 5] reported that none of the papillary lesions without atypia were associated with carcinoma. In the paper by Sydnor et al. [4], only 1 out of 13 (3%) excised benign papillomas was upgraded to malignancy at excision. While these four studies suggest that surgical excision is not necessary for percutaneously diagnosed benign papillary lesions, they were limited to the sample size of 8 to 38 patients. 

The studies from institutions with the larger sample sizes, including our own, are in consensus that papillary lesions should be excised for complete pathologic diagnosis as their results show an increased risk of coexistent carcinoma upon surgical excision. Results from Skandarajah et al. in 2007 and Rizzo et al. in 2008 had sample sizes of 86 and 80, respectively and supported excision of benign bland papillomas. Perhaps the most convincing data is from Skandarajah et al., who reported that 26 out of 80 (33%) papillary lesions without atypia were associated with upgrade to atypia or malignancy at surgical excision. This data suggested that one out of three patients undergoing core needle biopsy alone would have missed the diagnosis of atypia or cancer. 

With a sample size of 67 patients, we have one of the largest sample sizes in the literature that specifically target upgrade rates from benign papillomas found at percutaneous core biopsy. Our results demonstrate an upgrade rate of 19% including 7% upgrade to malignancy. These findings suggest that one out of five patients undergoing percutaneous core biopsy alone will be misdiagnosed.

The limitations of our study include the small sample size, which is however one of the largest sample sizes among published papers on the same topic. In addition, the patients with upgraded lesions on surgical excision were not followed to assess for the clinical outcome. While we assume that there is clinical benefit in diagnosing cancer, which would have been missed with the percutaneous core biopsy alone, it is uncertain whether surgical excision of those lesions which were upgraded to atypia actually changed morbidity and mortality.

## 4. Conclusion

Given that we have one of the largest samples sizes in the published literature, our results lend significant support to the existing data which suggests excision of benign bland papillary lesions diagnosed on percutaneous core biopsy.

## Figures and Tables

**Table 1:** Final pathology of 67 lesions following surgical excision.

Final surgical pathology	Number of lesions	Percentage (%)
Total	67	100
Benign	54	81
Cancer	5	7
DCIS	2	3
Invasive ductal carcinoma	3	4
High-risk lesions	8	12
ADH	6	9
ALH	1	1.5
LCIS	1	1.5

**Table 2:** Summary of literature: Benign papillary lesions diagnosed with core needle biopsy and followed up with surgical excision.

Year	Refererence	Sample size	Excisional pathology				
			Benign	Upgrade	Malignant	Atypia	Final recommendation
2011	Our work	67	54 (81%)	13 (19%)	5 (7%)	8 (12%)	Excision
2009	[2]	29	28 (97%)	1 (3%)	1 (3%)	0 (0%)	No excision
2008	[15]	48	38 (79%)	10 (21%)	7 (15%)	3 (6%)	Excision
2008	[6]	86	65 (75%)	21 (25%)	9 (10%)	12 (14%)	Excision
2007	[8]	80	54 (67.5%)	26 (32.5%)	15 (18.8%)	11 (14%)	Excision
2007	[4]	38	37 (97%)	1 (2.6%)	1 (2.6%)	0 (0%)	No excision
2007	[16]	20	13 (65%)	13 (65%)	7 (35%)	4 (20%)	Excision
2007	[12]	36	30 (84%)	5 (14%)	5 (14%)	0 (0%)	Excision
2006	[10]	25	20 (80%)	5 (20%)	5 (20%)	0 (0%)	Excision
2006	[17]	36	14 (39%)	10 (28%)	2 (5.5%)	8 (22%)	Excision
2006	[13]	37	32 (86%)	5 (14%)	5 (14%)	0 (0%)	No excision
2004	[14]	6	6 (100%)	0 (0%)	0 (0%)	0 (0%)	No excision
2004	[11]	13	9 (69%)	4 (31%)	2 (15.5)	2 (15.5%)	Excision
2004	[5]	8	8 (100%)	0 (0%)	0 (0%)	0 (0%)	No excision
2004	[3]	11	11 (100%)	0 (0%)	0 (0%)	0 (0%)	No excision
2003	[18]	31	29 (94%)	2 (6%)	2 (6%)	0 (0%)	Excision
2002	[19]	14	11 (78%)	3 (21%)	1 (7%)	2 (14%)	No excision
